# Systems biology analysis identifies *TNFRSF9* as a functional marker of tumor-infiltrating regulatory T-cell enabling clinical outcome prediction in lung cancer

**DOI:** 10.1016/j.csbj.2021.01.025

**Published:** 2021-01-21

**Authors:** Jae-Won Cho, Jimin Son, Sang-Jun Ha, Insuk Lee

**Affiliations:** a Department of Biotechnology, College of Life Science & Biotechnology, Yonsei University, Seoul 03722, Republic of Korea; b Department of Biochemistry, College of Life Science & Biotechnology, Yonsei University, Seoul 03722, Republic of Korea

**Keywords:** Tumor-infiltrating regulatory T cell, Tumor microenvironment, Functional markers, Coregulatory network, Context associated hub

## Abstract

Regulatory T cells (Tregs) are enriched in the tumor microenvironment and play key roles in immune evasion of cancer cells. Cell surface markers specific for tumor-infiltrating Tregs (TI-Tregs) can be effectively targeted to enhance antitumor immunity and used for stratification of immunotherapy outcomes. Here, we present a systems biology approach to identify functional cell surface markers for TI-Tregs. We selected differentially expressed genes for surface proteins of TI-Tregs and compared these with other CD4^+^ T cells using bulk RNA-sequencing data from murine lung cancer models. Thereafter, we filtered for human orthologues with conserved expression in TI-Tregs using single-cell transcriptome data from patients with non-small cell lung cancer (NSCLC). To evaluate the functional importance of expression-based markers of TI-Tregs, we utilized network-based measure of context-associated centrality in a Treg-specific coregulatory network. We identified *TNFRSF9* (also known as *4-1BB* or *CD137*), a previously reported target for enhancing antitumor immunity, among the final candidates for TI-Treg markers with high functional importance score. We found that the low *TNFRSF9* expression level in Tregs was associated with enhanced overall survival rate and response to anti-PD-1 immunotherapy in patients with NSCLC, proposing that TNFRSF9 promotes immune suppressive activity of Tregs in tumor. Collectively, these results demonstrated that integrative transcriptome and network analysis can facilitate the discovery of functional markers of tumor-specific immune cells to develop novel therapeutic targets and biomarkers for boosting cancer immunotherapy.

## Graphical abstract

**Figure:**
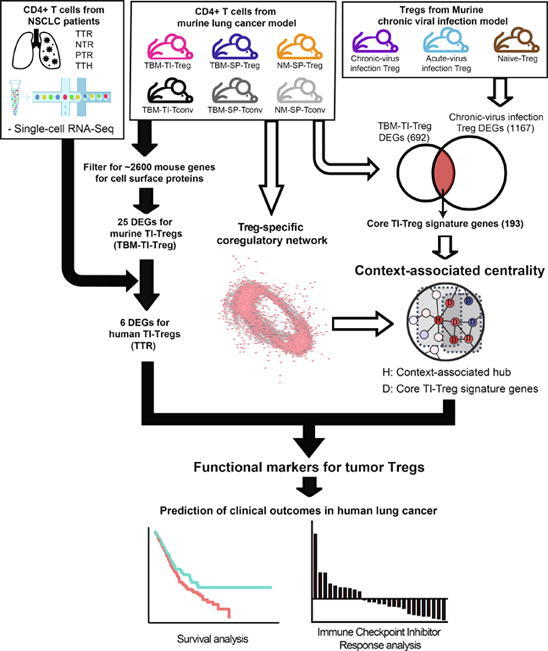

## 1 Introduction

Lately, immune checkpoint inhibitors are widely used for antitumor treatment owing to their beneficial therapeutic efficacy and long-term duration effect [1], [2]. Thus, the better understanding of cancer–immune interactions in the tumor microenvironment (TME) will improve the efficacy of cancer immunotherapy [3]. Regulatory T cells (Tregs) suppress tumor immune surveillance by inhibiting effector T cell responses [4], [5]. In addition, Tregs are preferentially enriched in the TME to sustain immunotolerance for cancer cells [5], [6], [7], [8], [9], [10]. Tumor-infiltrating Tregs (TI-Tregs) express immune checkpoint molecules such as CTLA-4, PD-1, TIM-3, LAG3, and TIGIT [11], [12], [13], [14]. Therefore, TI-Tregs are considered major cellular targets for cancer immunotherapy [10], [13], [15], [16], [17]. In addition, novel cell surface proteins specific for TI-Tregs would provide new opportunities to modulate the antitumor activity of immune cells in TME. For example, antibody blockade against TI-Treg-specific receptors that promote immune suppressive activity will enhance the clinical outcome of antitumor treatments. CD25 is a surface protein commonly used as a marker for Tregs [7], [8], [18], [19], [20]. However, inhibition of normal Tregs would lead to severe autoimmunity during cancer immunotherapy [21]. Therefore, target specificity of the TI-Treg marker is a critical factor for its therapeutic purpose [22], [23], [24]. In the present study, we performed systems biology analysis across bulk and single-cell transcriptome profiles from multiple species and network-based analysis to identify functional surface proteins specific for TI-Tregs. We retrieved TNFRSF9, whose inhibition was reported to increase anti-tumor immunity [25], [26], among the final candidates for surface marker proteins. Our result also suggests that other candidate proteins are also likely to be useful therapeutic targets that modulate TI-Treg activity. Notably, for the first time, we demonstrated that *TNFRSF9* expression level in the TI-Tregs can predict response to anti-PD-1 immunotherapy in human non-small cell lung cancer (NSCLC), which verifies functional impact of *TNFRSF9* in immune–tumor interactions. These results demonstrate feasibility of identification of functional markers for tumor-reactive immune cells to facilitate development of novel therapeutic targets and biomarkers for improving cancer treatment via modulating immune–tumor interactions.

## 2 Material and methods

### 2.1 Differential expression (DE) analysis for mouse bulk RNA-seq data

We used bulk RNA sequencing (RNA-seq) data for CD4^+^ T cells of the murine lung cancer model generated during our previous study [9]. This provided transcriptome profiles for six different CD4^+^ T cell populations: tumor-infiltrating Treg from tumor-bearing mouse (TBM-TI-Treg) and conventional CD4^+^ T cell from tumor-bearing mouse (TBM-TI-Tconv), spleen-derived Treg from tumor-bearing mouse (TBM-SP-Treg) and those from normal mouse (NM-SP-Treg), spleen-derived conventional CD4^+^ T cell from tumor-bearing mouse (TBM-SP-Tconv) and those from normal mouse (NM-SP-Tconv) (available from GSE120280 of Gene Expression Omnibus [27]). Approximately 2,600 mouse genes for cell surface proteins were compiled through gene filtration from the Consensus Coding Sequence (CCDS) database [28] for “cell membrane and cell surface” proteins annotated using Swiss-Prot [29] and for “external side of plasma membrane” proteins annotated via Gene Ontology (GO) [30] with only experimental evidence (represented by evidence codes of IDA, IPI, IMP, IGI, IEP, TAS and EXP). Sequence reads of mouse RNA-seq data were aligned to the mouse reference genome (UCSC mm10) [31] using STAR-2.5.2a [32], and subsequently quantified based on FeatureCounts [33]. DE analysis was performed using DESeq2 [34] and Characteristic Direction [35] analyses. For DESeq2 analysis, we selected genes with fold change (FC) > 2 and *P* < 0.01 as candidates. For Characteristic Direction analysis, genes were selected as candidates if their characteristic scores were higher than that of the uniform model ($\sqrt{1/n}$; n = number of genes). Only genes with positive characteristic scores were selected to obtain induced genes in the tumor. For evaluation of candidate mouse genes in human cancer data, we identified their human orthologous proteins based on bidirectional best hits using basic local alignment search tool [36].

### 2.2 Human scRNA-seq data analysis

Single-cell RNA sequencing (scRNA-seq) data for CD4^+^ T cells from patients with NSCLC were obtained from a previous study (available from GSE99254) [37]. For scRNA-seq data analysis, we used presorted T cells from the original studies (TTR: tumor Treg, NTR: normal Treg, PTR: PBMC Treg, TTH: tumor Tconv) without additional annotation work. For DE analysis, DEsingle [38] was performed to compare TTR and three other cell types (TTR vs NTR, TTR vs PTR, TTR vs TTH). All differentially expressed genes (DEGs) were selected if they had an FC > 2 and *P* < 0.01.

### 2.3 Mouse microarray data analysis

Microarray data of lymphocytic choriomeningitis virus (LCMV)-infected Treg cells were obtained from a previous study (available from GSE63876) [39] for mouse Tregs with chronic virus infection, those with acute virus infection, and naïve Tregs. DE analysis for microarray data was performed using the Limma package [40] and Characteristic Direction analysis [35]. The same criteria for DEG selections were employed for mouse RNA-seq data analysis. DEGs specific for Tregs with chronic virus infection were obtained by subtracting DEGs for acute Tregs vs naive Tregs from DEGs for chronic Tregs vs naive Tregs. We defined core TI-Treg signature genes by using the intersection between DEGs for TBM-TI-Treg of the mouse lung cancer model and DEGs for Tregs with chronic virus infection of mouse.

### 2.4 Construction of the Treg-specific coregulatory network

Genes with similar expression patterns tend to be regulated together and are functionally associated [41]. Thus, coregulatory relationships can indicate functional coupling of two genes in certain cellular contexts. We inferred functionally associated genes in the context of Tregs based on the correlation of gene expression across the three distinct populations of mouse Tregs from the mouse lung cancer model using RNA-seq data (TBM-TI-Treg, TBM-SP-Treg, NM-SP-Treg). To avoid false positive links by technical variance, we filtered out genes whose maximum expression levels across samples were lower than those for genes expected not to express in T cells, such as marker genes for B cells (*CD19*, *CD79A*, *CD79B*) and macrophages (*CD163*, *CD14*, *CSF1R*). Finally, expression values of 4,446 genes were root mean square (RMS) normalized and applied to Pearson’s correlation coefficient (PCC) analysis to infer coexpression links. By only using links with a significant PCC (*P* < 0.01), we finally obtained the Treg-specific coregulatory network composed of 924,319 links among 4,427 genes. To evaluate the quality of the network, we assessed the network modularity of genes involved in Treg functions for autoimmunity using within-group connectivity significance. We compiled genes associated with rheumatoid arthritis, psoriasis, and multiple sclerosis genes, often differentially expressed in Tregs, from the DisGeNET database [42]. We measured significance of connectivity for each group of disease genes in the Treg-specific network based on the null network model of 100,000 random links for the same group size.

### 2.5 Network-based measure of context-associated centrality

The functional importance of genes for a relevant cellular context can be assessed by integration of the functional gene network with context-associated genes. In this approach, functional centrality of each gene for the given biological context is evaluated by overrepresentation of its connected network neighbors in DEGs associated with the context of interest using Fisher’s exact test, resulting in context-associated centrality (CAC) scores. For the list of genes associated with the context of TI-Treg, we used core TI-Treg signature genes which are the intersection of DEGs for TBM-TI-Treg from the mouse lung cancer model and DEGs for Treg from the mouse chronic virus infection model. We finally calculated CAC scores with the Treg-specific network and core TI-Treg signature genes.

### 2.6 Survival analysis

RSEM normalized expression data of lung adenocarcinoma (LUAD) and lung squamous cell carcinoma (LUSC) were obtained from The Cancer Genome Atlas (TCGA) (https://gdac.broadinstitute.org/). Survival data were obtained from TCGA Pan-Cancer Clinical Data Resource (TCGA-CDR) [43]. The expression level of *TNFRSF9* was normalized for the amount of Treg using the expression level of *FOXP3*. Patients were labeled as “High” or “Low” when the normalized expression level of *TNFRSF9* was within the top 30% or below the bottom 30%, respectively. Kaplan-Meier analysis was performed for overall survival.

## 3 Results

### 3.1 Overview of systems biology approach to identify functional markers of TI-Tregs

**Fig. 1:**
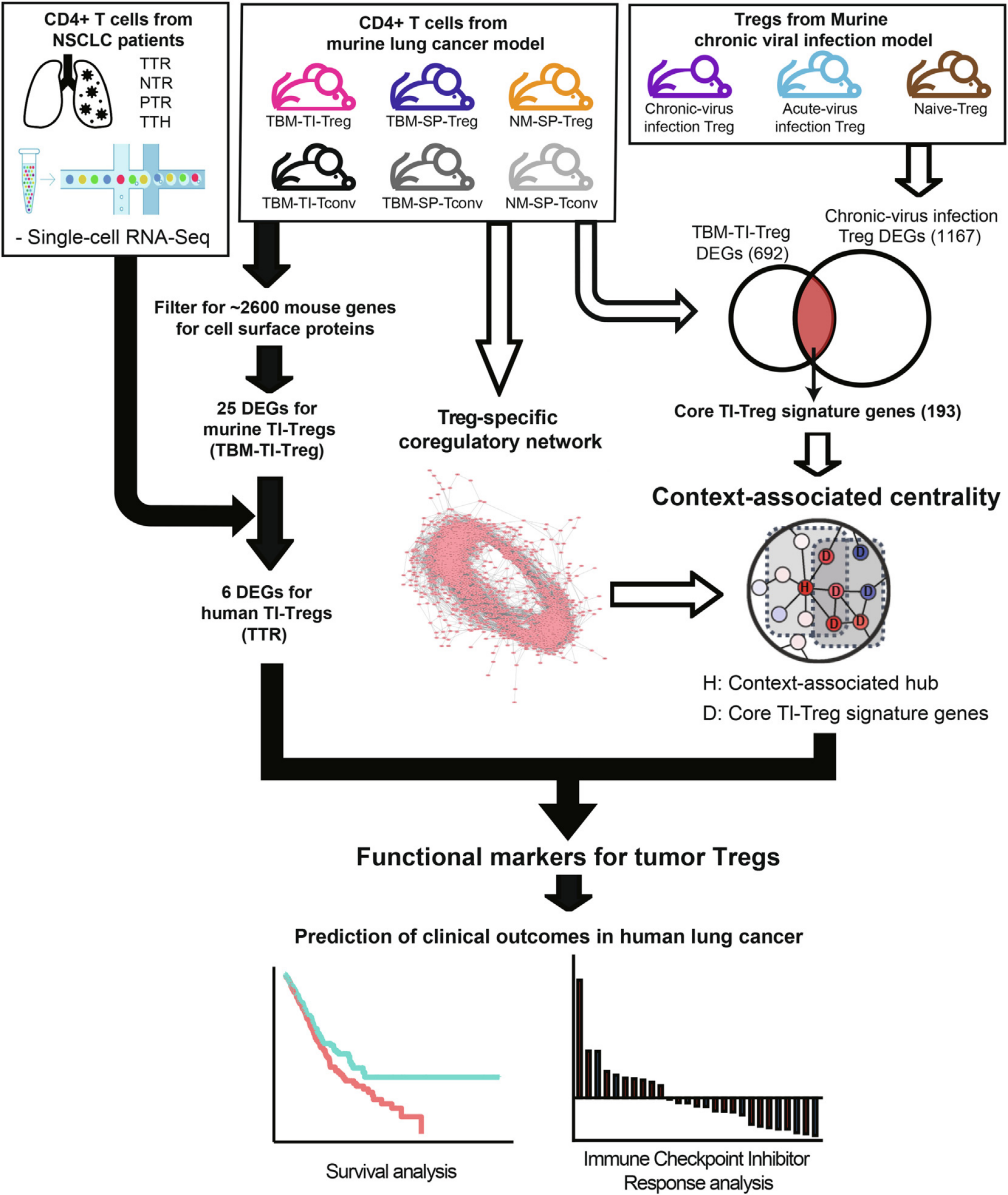
Schematic overview of a systems biology approach to identify functional marker genes of tumor-infiltrating regulatory T cells (TI-Tregs).

We used a systems biology approach to identify functional markers of a tumor-specific immune cell type, TI-Treg, based on the integrative analysis of three sets of CD4^+^ T cell transcriptome profiles from distinct biological contexts (Fig. 1) [44]. First, we identified DEGs for surface proteins of TI-Tregs using bulk transcriptome data from the mouse lung cancer model. Second, we filtered expression-based markers from the mouse lung cancer model for human DEGs for TI-Tregs using single-cell transcriptome data from patients with NSCLC. Third, we evaluated the functional importance of expression-based markers for TI-Tregs based on context-associated network centrality, in which bulk transcriptome data from mouse Tregs with chronic virus infection were used to define core signature genes for TI-Treg context.

Majority of orthologous genes between mouse and human have conserved expression in immune cells [45]. Because of high experimental accessibility to the mouse system, it would be advantages to obtain candidate genes conserved between mouse and human for the follow-up studies. In addition, different technologies of gene expression measurement tend to complement each other, improving both sensitivity and specificity of expression-based gene prioritization [46]. Therefore, we combined candidate genes generated with transcriptome data of tumor-associated immune cell obtained from different organisms, mouse and human, along with different technical platforms, bulk RNA-seq, single-cell RNA-seq and microarray. Marker genes that play key roles in a specific immune cell type in a specific biological context, such as tumor tissue, would be promising therapeutic targets. Functional importance does not correlate with expression change in the relevant biological context, because many effector genes downstream of the regulatory hierarchy, rather than upstream key regulators, often show larger expression changes during transition to the context. Thus, we harnessed network-based measures of functional importance, which not only account for the expression changes of target genes of interest but also those of other genes functionally connected to the target genes in the network. Network-based reinterpretation of gene expression data was found useful in prioritization of essential genes [47] and disease genes [48], and drug repositioning [49].

### 3.2 Identifying marker genes for cell surface proteins of TI-Tregs in mouse

**Fig. 2:**
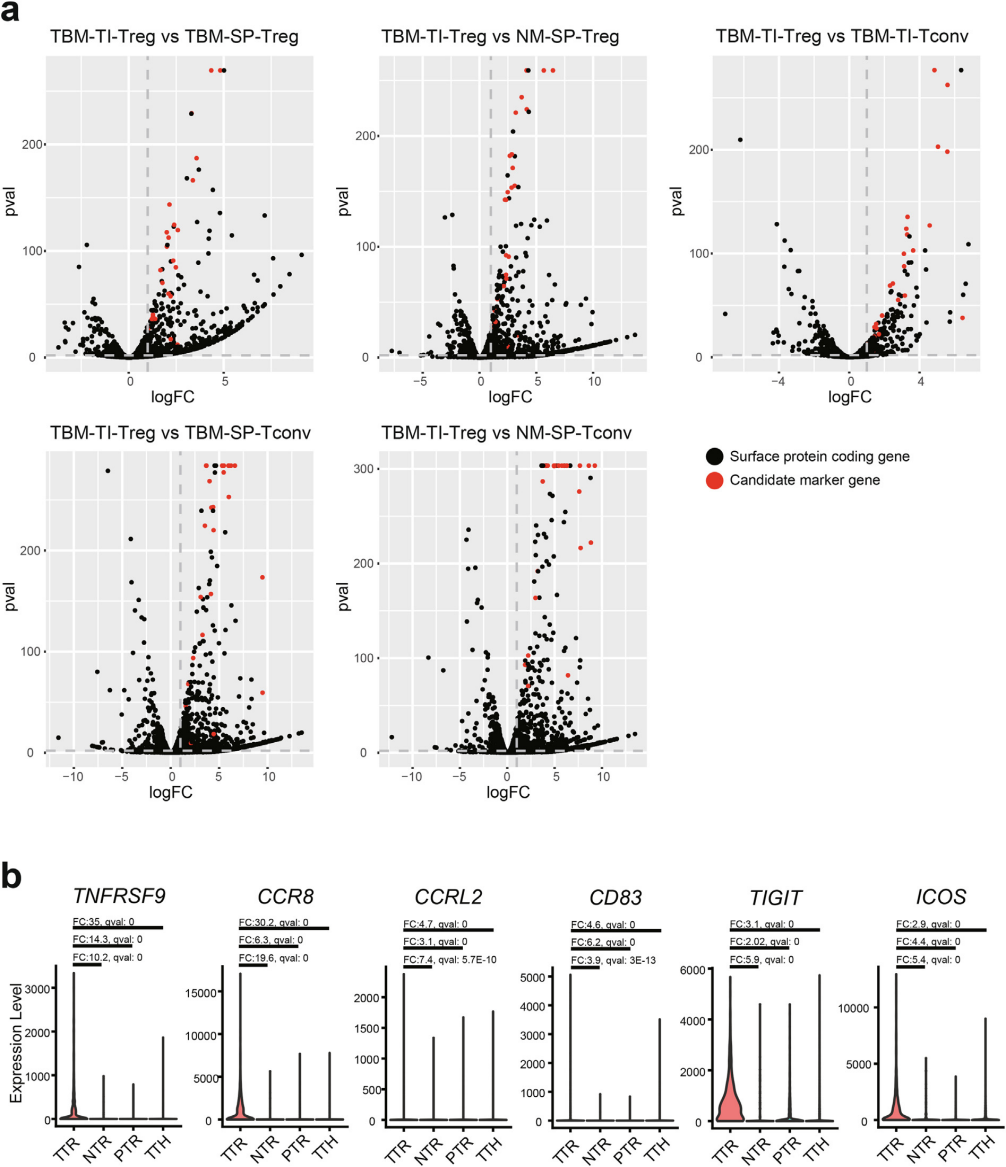
Expression-based marker genes of TI-Tregs in mouse and human. (a) Volcano plots that show differential expression analysis from comparisons of tumor-bearing mouse TI-Treg (TBM-TI-Treg) with five other CD4^+^ T cell types in different tissue contexts: conventional CD4^+^ T cell from tumor-bearing mouse (TBM-TI-Tconv), spleen-derived Treg from tumor-bearing mouse (TBM-SP-Treg) and those from normal mouse (NM-SP-Treg), spleen-derived conventional CD4^+^ T cell from tumor-bearing mouse (TBM-SP-Tconv) and those from normal mouse (NM-SP-Tconv). The final 25 candidate markers by intersection of the comparisons are marked in red. (b) Violin plots that show the expression of six human genes differentially expressed in tumor Treg (TTR), normal Treg (NTR), PBMC Treg (PTR), tumor Tconv (TTH). (For interpretation of the references to colour in this figure legend, the reader is referred to the web version of this article.)

The first requirement for the marker gene is a noteworthy change in expression specific for the target cellular context. For this study, we tested DE for TI-Tregs compared with other CD4^+^ T cells. If marker proteins are expressed on the cell surface, modulation of cellular functions by antibody-based agonists or antagonists would provide opportunities for therapeutic applications. Therefore, we focused on approximately 2,600 mouse genes encoding cell surface proteins (Material and Methods) in our search for TI-Treg marker genes. We performed two complementary DE analysis methods, DESeq2 and Characteristic Direction [34], [35], to increase the reliability of candidate genes. While DESeq2 performs gene-by-gene DE analysis, Characteristic Direction considers overall expression profiles. DEGs specific for TI-Tregs were obtained by comparing their expression profiles with those of five other CD4^+^ T cell populations derived from various tissue contexts, TBM-TI-Treg compared with TBM-TI-Tconv, TBM-SP-Treg, TBM-SP-Tconv, NM-SP-Treg, and NM-SP-Tconv (Fig. 2a), resulting in five sets of DEGs. As two complementary DE analysis methods were used, 10 sets of DEGs were obtained in total. We used the intersection of the 10 sets of DEGs, resulting in 25 candidate marker genes (Table S1). We retrieved well-known immune checkpoint molecules, *Pdcd1, Ctla4, Tigit, Cd74,* and *Icos* [13], [50], [51], [52], [53], [54] and *Ccr8*, a previously reported chemokine receptor for TI-Tregs [8], among the 25 candidate markers. This suggests that the other candidate genes are also likely associated with immune–tumor interactions.

### 3.3 Filtration for expression-based markers for TI-Tregs conserved in human

Systematic comparison of global gene expression profiles of immune cells between mouse and human demonstrated that although the expression patterns of most orthologous genes between both these immune systems were highly conserved, several hundred genes showed clearly divergent expression between them [45]. Therefore, we needed to validate markers of mouse immune cells in human counterparts for future clinical application. To verify the 25 expression-based marker genes from the mouse lung cancer model in the human tumor, we utilized single-cell transcriptome data for CD4^+^ T cells from patients with NSCLC [37]. We performed DE analysis using DEsingle [38] for presorted and annotated T cells from the original studies (TTR: tumor Treg, NTR: normal Treg, PTR: PBMC Treg, TTH: tumor Tconv). To identify DEGs for TI-Tregs, we compared TTR with three other cell types (TTR vs NTR, PTR, and TTH). We found six of the candidates, *TNFRSF9, CCR8, CCRL2, CD83, TIGIT, ICOS*, differentially expressed in TTR compared with all other cell populations in human NSCLC (Fig. 2b). Thus, we conclude that these six validated markers that showed conserved DE specific for TI-Tregs can be used for clinical applications.

### 3.4 Coregulatory gene network for measuring functional importance of markers

Expression-based marker genes of tumor-associated Tregs are not necessarily key players in Treg functions in the tumor. To validate the functional importance of expression-based marker genes, we employed a network-based measure of functional importance, network centrality. The functional importance of several genes depends on cellular context. Thus, we needed to measure network centrality, accounting for context-specific information available from context-associated expression signatures. Therefore, we calculated the CAC of a gene based on enrichment of context-associated signature genes among functionally connected genes to the target gene in the network.

**Fig. 3:**
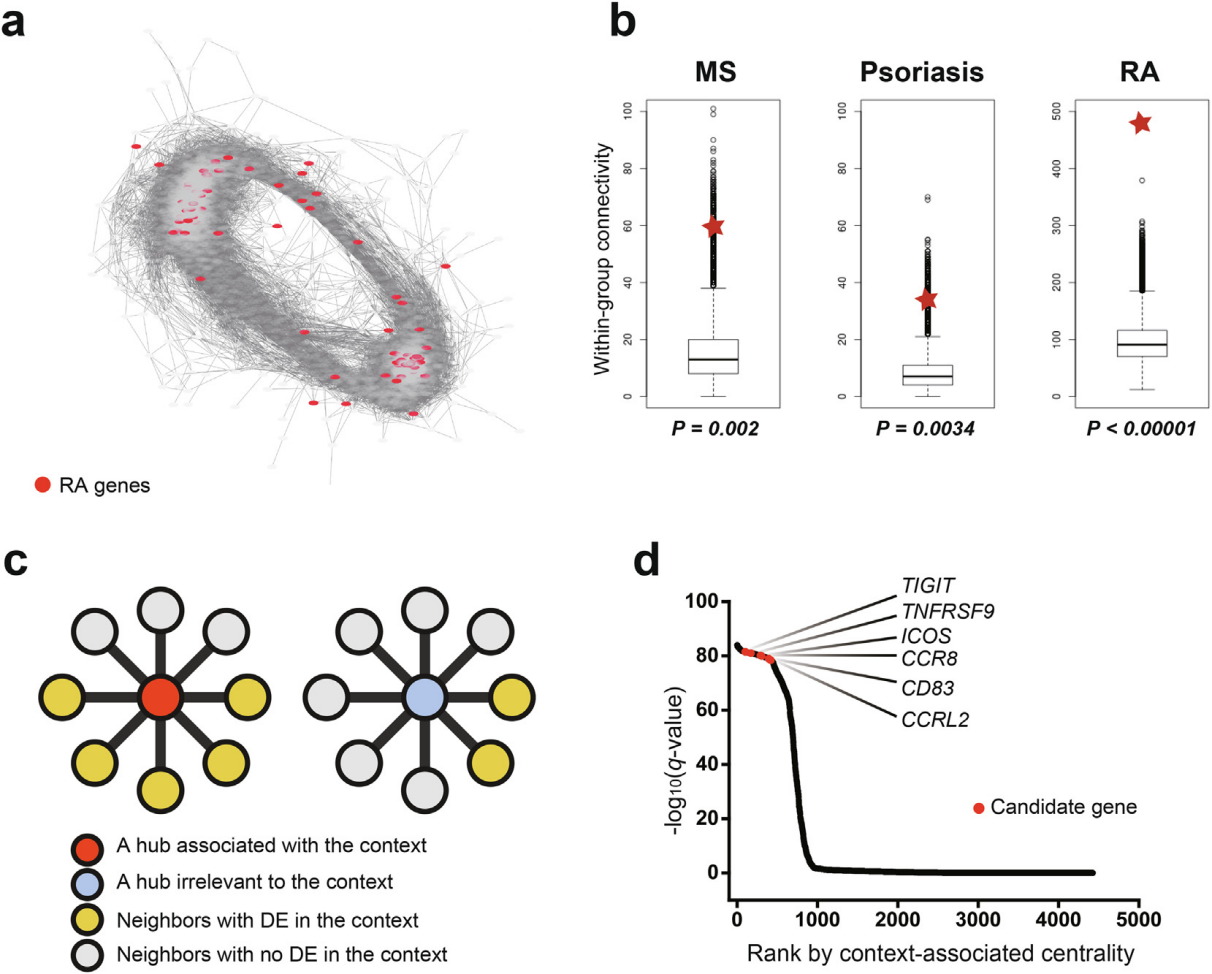
Network-based prioritization of functional markers of tumor-infiltrating regulatory T cells (TI-Tregs). (a) Layout of coregulatory network for Tregs (Treg-specific network). Genes associated with rheumatoid arthritis (RA) are represented as red nodes. (b) Within-group connectivity for each group of genes associated with multiple sclerosis (MS), psoriasis, and RA. Within-group connectivity by Treg-specific network and read disease gene sets are represented as red stars. (c) Illustrative summary of context-associated centrality (CAC). (d) Genes sorted by CAC score (-log_10_(*q-value*) of enrichment of network neighbors among context-associated signature genes) and six expression-based markers in human TI-Tregs are represented as red points. (For interpretation of the references to colour in this figure legend, the reader is referred to the web version of this article.)

As marker genes that play important roles in Treg function were sought, a network of genes specific for Tregs was appropriate for the analysis. To construct such a network, we utilized bulk RNA-seq profiles for Tregs from three distinct tissue contexts of the murine lung cancer model (TBM-TI-Treg, TBM-SP-Treg, NM-SP-Treg). We inferred coregulatory links based on the coexpression of gene pairs across bulk RNA-seq profiles and obtained a network comprising 924,319 links among 4,427 genes by using links with a significant PCC (*P* < 0.01) (Fig. 3a). Treg anomalies cause wide spectrum of autoimmune diseases [55]. There may exist a group of genes for Treg function and homeostasis, which are expected to collaborate closely with each other in Treg. Therefore, we assessed the quality of the Treg-specific network based on the modularity of genes involved in the autoimmune diseases such as rheumatoid arthritis, psoriasis, and multiple sclerosis, which were compiled from the DisGeNET database [42]. We found that within-group connectivity for all three disease gene sets was significantly higher compared with the null model (*P* < 0.00001, 0.00316, and 0.0019, respectively; Fig. 3b). In contrast, genes associated with autism (a neuropsychological disorder), type 2 diabetes (a metabolic disease) and cancer did not show significantly higher within-group connectivity than null model in the Treg-specific network (Fig. S1). These results together suggest that the constructed Treg-specific network will provide reasonable quality for follow-up network-based analysis.

### 3.5 TI-Treg markers have high functional importance

Thereafter, we acquired a list of signature genes associated with TI-Tregs. All DEGs for TBM-TI-Treg from the murine lung cancer model could have been used; however, they also carry genes irrelevant to TI-Tregs. Thus, we filtered DEGs for TBM-TI-Treg for another set of DEGs from Tregs with tumor-like condition, chronic virus infection [56]. Previous studies demonstrated similar transcriptional features of tumor-infiltrating CD8^+^ T cells to those of cells with chronic virus infection [57], [58], implying that CD4^+^ T cells may also show similar transcriptional responses to the tumor and chronic virus infection. Thus, their overlap will provide a core gene signature of tumor-specific Tregs. We compiled an expression signature of chronic virus-specific Tregs from microarray-based transcriptome profiles for murine Tregs isolated from naïve and infected mice with chronic (LCMV CL13) or acute virus (LCMV Armstrong) [39]. We subsequently identified DEGs for Tregs with chronic virus infection by comparison with those with acute virus infection and no infection. Thus, 193 DEGs (152 upregulated and 41 downregulated, Table S2-3) overlapped with 692 DEGs from tumor-specific murine Tregs and 1,167 DEGs from murine Tregs infected with the chronic virus, which indicates a significant association of transcriptional features between tumor and chronic virus infection contexts as we hypothesized (*P* = 1.32E-81 by Fisher’s exact test).

Finally, genes of the Treg-specific network were ranked by CAC, which was distinct from normal degree centrality measure based on the entire set of connected neighbors in the network. In CAC, a hub gene with a more substantial overlap between network neighbors and context-associated signature genes was ranked higher in the prioritized candidate list (Fig. 3c). Notably, all six expression-based markers for human TI-Tregs, *TIGIT, TNFRSF9, ICOS, CCR8, CD83,* and *CCRL2*, were ranked within the top 10%, which indicates that they are likely to play major roles in tumor-associated Treg functions (Fig. 3d, Table S4-5). These results also suggest that antibody-mediated stimulation or inhibition of these surface markers can modulate Treg functions in the tumor, potentially leading to changes in immune–tumor interactions.

The successful prioritization of functional markers for TI-Tregs may depend on TI-Treg-associated signature genes and network links specific for Tregs. To test whether using a cell-type specific network is critical, we performed the same gene prioritization analysis with an integrated functional gene network for mouse, MouseNet (v2) [59]. Notably, all six markers for human TI-Tregs had no connection to context-associated signature genes in MouseNet. This indicates that gene prioritization by CAC works accurately with coregulatory networks for relevant cell types, rather than a highly comprehensive and integrated functional network.

### 3.6 *TNFRSF9* levels in tumor Tregs can predict clinical outcome in human lung cancer

**Fig. 4:**
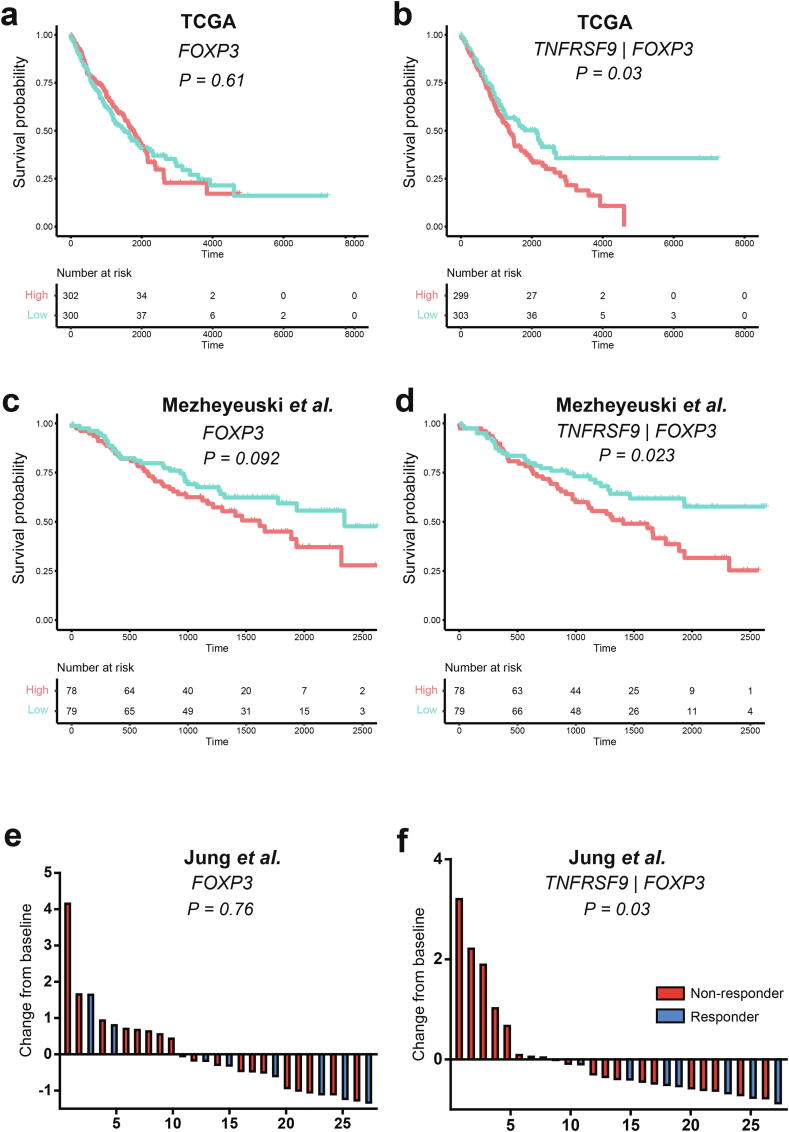
*TNFRSF9* expression level in tumor-infiltrating regulatory T cells (TI-Tregs) is predictive of clinical outcomes in anticancer treatment. (a-b) Kaplan-Meier analysis curves by high (top 30%) and low (bottom 30%) expression levels of *FOXP3* (a), expression level of *TNFRSF9* normalized by that of *FOXP3* (b) in tumor samples from The Cancer Genome Atlas-lung adenocarcinoma (TCGA-LUAD) and TCGA-lung squamous cell carcinoma (LUSC). (c-d) Kaplan-Meier analysis curves by high (top 40%) and low (bottom 40%) expression levels of *FOXP3* (a), expression level of *TNFRSF9* normalized by that of *FOXP3* (b) in tumor samples from Swedish cohort of lung cancer (GSE81089) (e-f) Waterfall plots showing expression changes from baseline (median) for *FOXP3* (e) and *TNFRSF9* normalized by *FOXP3* (f) in tumor samples from patients with non-small cell lung cancer (NSCLC) treated by anti-PD-1 inhibitors (GSE135222).

Among the six final candidates for TI-Treg functional markers, *TNFRSF9* is known as a Treg-specific gene [26]. Thus, we tested its potency as a biomarker for clinical outcome using bulk transcriptome profiles for tumor samples. As we identified markers from the murine lung cancer model and verified them in human lung cancer, we used bulk RNA-seq profiles for TCGA-LUAD and TCGA-LUSC samples along with their clinical annotations as validation data. For the expression level of *FOXP3* that is proportional to the amount of TI-Tregs in the tumor sample, no prediction power was observed for overall survival as expected (Fig. 4a). However, we found that the expression level of *TNFRSF9* normalized by that of *FOXP3* favored poor prognosis (Fig. 4b). This indicates that the expression of *TNFRSF9* in TI-Tregs negatively influences the prognosis of anticancer treatment. The observed prognostic prediction by the expression level of *TNFRSF9* in TI-Tregs was also validated in an independent cohort of patients with NSCLC recruited in Sweden (available from GSE81089) [60] (Fig. 4c-d). Despite of distinct tumor immune microenvironment across different cancer types [61], we wonder if the TI-Treg-specific expression level of *TNFRSF9* can predict prognosis in other cancer types. From the analysis on 34 distinct cancer types using data available in TCGA, we found that high TI-Treg-specific expression level of *TNFRSF9* could predict poor survival in four additional cancer types: glioma (GBMLGG), head and neck squamous cell carcinoma (HNSC), pancreatic adenocarcinoma (PAAD) and uveal melanoma (UVM) (Fig. S2). In addition, we tested whether the expression level of *TNFRSF9* in TI-Tregs correlates with stage of lung cancer and found that it was significantly higher in stage-4 patients compared with stage-1 patients (*P* = 0.03, Mann-Whitney *U* test) (Fig. S3).

Next, we tested the expression level of *TNFRSF9* in the prediction of response to anti-PD-1 immunotherapy. We used bulk RNA transcriptome data for tumor biopsies from a cohort of patients with NSCLC who underwent anti-PD-1 inhibitor treatment (available from GSE135222) [62]. We found that the expression level of *TNFRSF9* normalized by that of *FOXP3* inversely correlated with the response rate to anti-PD-1 immunotherapy, whereas the abundance of Tregs could not predict the anti-PD-1 response (Fig. 4e-f). We conducted similar analysis for two independent cohorts of melanoma patients with anti-PD-1 treatment [63], [64], but could not observe significant correlation between TI-Treg-specific expression level of *TNFRSF9* with the anti-PD-1 response rate. These results may be attributable to the distinct tumor immune microenvironment among different cancer types [61]. Therefore, we conclude that the expression level of *TNFRSF9* in TI-Tregs is a novel biomarker that can predict the prognosis of anticancer treatment and response to anti-PD-1 immunotherapy in human lung cancer.

## 4 Discussion

*TNFRSF9*, also known as *4-1BB*, was reportedly expressed in mouse and human TI-Tregs [25]. *TNFRSF9* was also reported as an activation marker for tumor-reactive Tregs [37]. In addition, the anticancer effect of anti-4-1BB treatment was reported in multiple types of mouse cancer such as thymoma, neuroblastoma, bladder cancer, glioblastoma, prostate cancer, and renal cancer models [25], [26]. Results from our study together with these previous reports confirm that *TNFRSF9* is associated with the activation of TI-Tregs, and their inhibition may boost anticancer treatments including lung cancer immunotherapy by reducing immune suppressive function of Tregs in tumor. However, *TNFRSF9* also acts as a costimulatory receptor for CD8^+^ T cells, thus an agonistic antibody for *TNFRSF9* is already under clinical trial. Therefore, direct targeting of *TNFRSF9* to suppress TI-Tregs should be practiced with care. Whether the use of 4-1BB blocking antibody is beneficial in controlling tumor growth or not might be dependent on the tumor microenvironment. For instance, in the immunosuppressive tumor microenvironment with a large population of Treg cells and relatively few CD8^+^ T cells, the use of 4-1BB blocking antibody will help induce overall anti-tumor immune activity. On the other hand, the use of 4-1BB agonistic antibody may be an effective treatment option in the immunogenic tumor microenvironment where the abundance of CD8^+^ T cells is relatively high compared to that of Treg cells. To the best of our knowledge, this is the first demonstration of the usefulness of *TNFRSF9* as a biomarker to predict the response to anti-PD-1 immunotherapy. Thus, this study expands the clinical application of *TNFRSF9* expression in TI-Tregs as a potential biomarker in anti-PD-1 cancer immunotherapy.

In the present study, we utilized network-based prioritization of genes with functional importance. The network-based gene prioritization approach has already proven useful in identification of genes associated with cancer and other human diseases [49], [65], [66], [67], [68]. We applied a similar network-based strategy to identify functional markers for particular types of immune cells associated with tumor context. We also found that network-based gene prioritization for specific cell types works accurately with the coregulatory network for relevant cell types, as shown in this study using a Treg-specific coregulatory network to prioritize functional marker genes of Tregs. In theory, cell-to-cell variance of single-cell transcriptome data enables inference of the cell type-specific coregulatory network [69]. However, there is a great challenge in single-cell network inference due to high levels of technical noise and sparsity in single-cell transcriptome data. Nevertheless, a single-cell gene regulatory network will be useful in generalizing our approach to identify functional markers of other immune cell subsets in various disease contexts.

## CRediT authorship contribution statement

**Jae-Won Cho:** Methodology, Software, Investigation, Data curation, Writing - original draft, Writing - review & editing. **Jimin Son:** Investigation, Resources, Data curation, Writing - review & editing. **Sang-Jun Ha:** Conceptualization, Methodology, Investigation, Writing - review & editing, Supervision, Project administration, Funding acquisition. **Insuk Lee:** Conceptualization, Methodology, Investigation, Writing - original draft, Writing - review & editing, Supervision, Project administration, Funding acquisition.

## Declaration of Competing Interest

The authors declare that they have no known competing financial interests or personal relationships that could have appeared to influence the work reported in this paper.
