# Supplementary material for: Systems biology analysis identifies TNFRSF9 as a functional marker of tumor-infiltrating regulatory T-cell enabling clinical outcome prediction in lung cancer
Source: Comput Struct Biotechnol J. 2021 Jan 21;19:860–8. doi: 10.1016/j.csbj.2021.01.025 (PMC7851794; doi:10.1016/j.csbj.2021.01.025)
Supplement: Supplementary data 1 [file mmc1.docx]

***Supplemental Online Information***

**Systems biology analysis identifies *TNFRSF9* as a functional marker of tumor-infiltrating regulatory T-cell enabling clinical outcome prediction in lung cancer**

Jae-Won Cho, Jimin Son, Sang-Jun Ha and Insuk Lee

**Contents**

**Fig. S1. Within-group connectivity for each group of genes associated with autism, type 2 diabetes (T2D) and cancer.**

**Fig. S2. Kaplan-Meier analysis curves by high and low expression levels of *TNFRSF9* normalized by that of *FOXP3* in tumor samples from The Cancer Genome Atlas**

**Fig. S3. Expression level of *TNFRSF9* in TI-Tregs among lung cancer patients at different stage (1-4).**


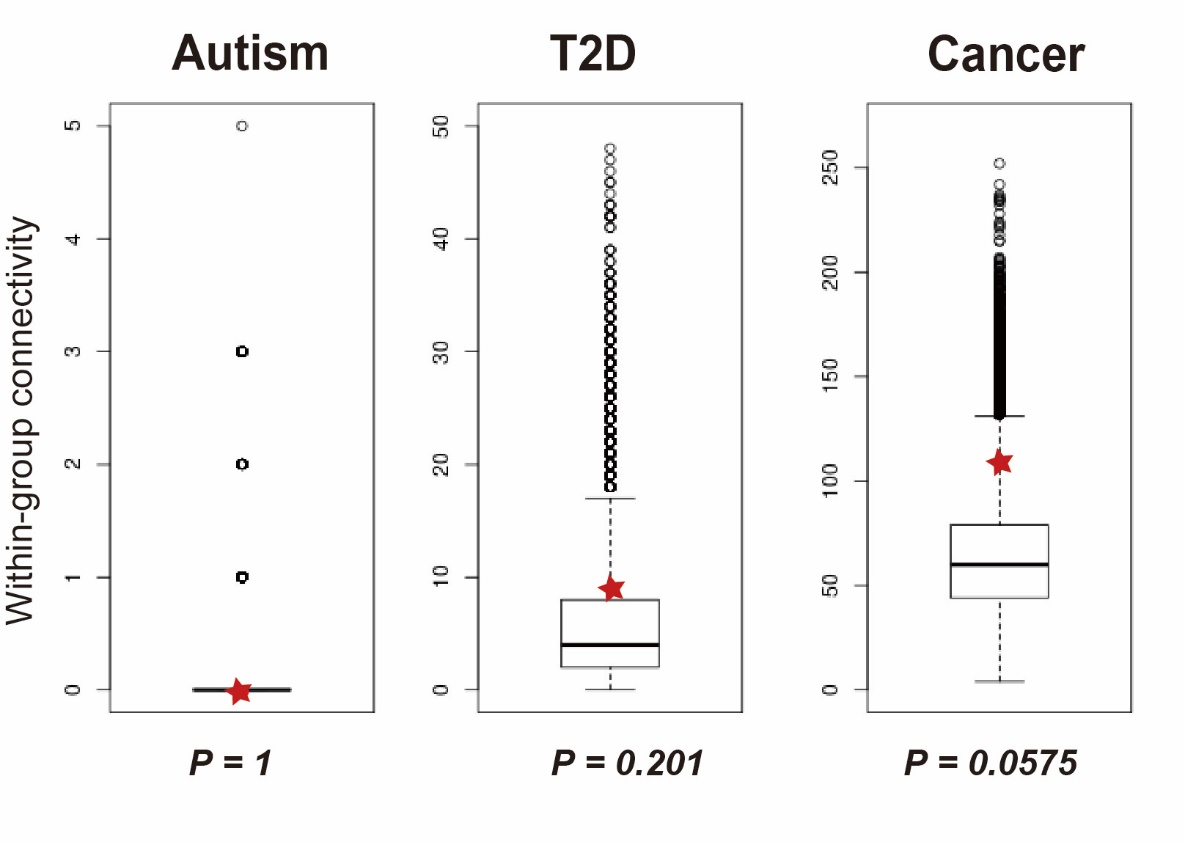


**Fig. S1.** **Within-group connectivity for each group of genes associated with autism, type 2 diabetes (T2D) and cancer.** Within-group connectivity by Treg-specific network and disease gene sets are represented as red stars.


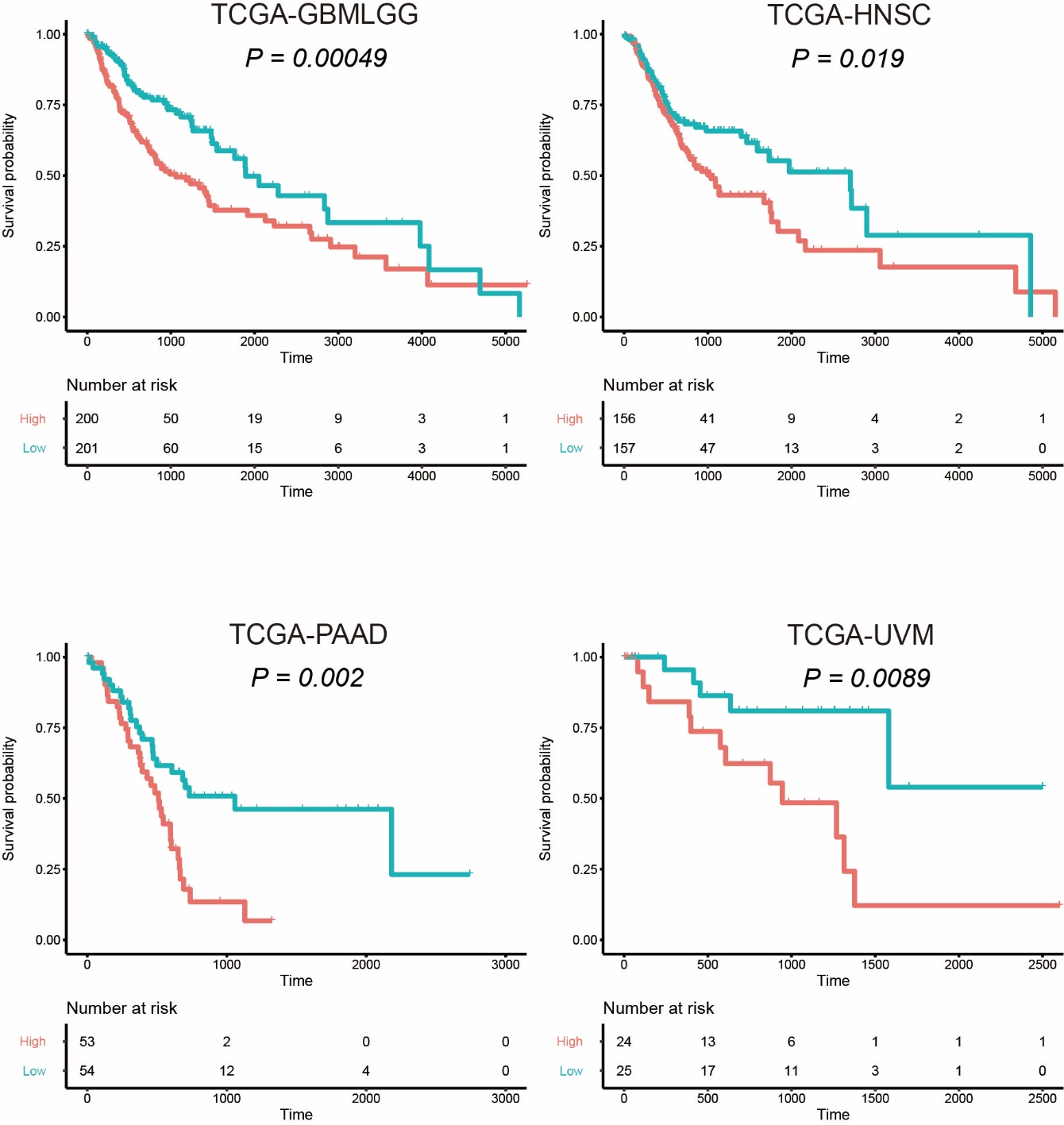


**Fig. S2.** **Kaplan-Meier analysis curves by high and low expression levels of *TNFRSF9* normalized by that of *FOXP3* in tumor samples from The Cancer Genome Atlas:** glioma (TCGA-GBMLGG), head and neck squamous cell carcinoma (HNSC), pancreatic adenocarcinoma (PAAD) and uveal melanoma (UVM)


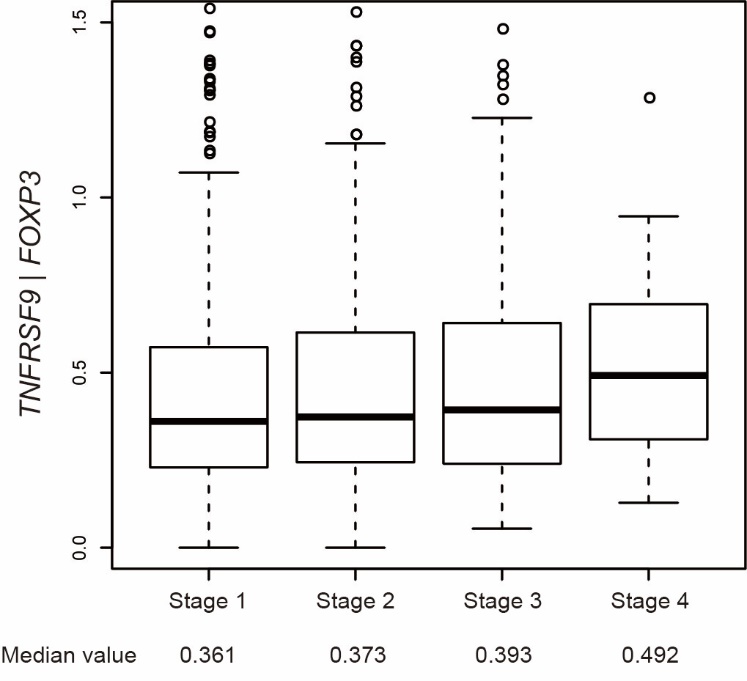


**Fig. S3. Expression level of *TNFRSF9* in TI-Tregs among lung cancer patients at different stage (1-4).** Expression level of *TNFRSF9* in TI-Tregs was significantly higher in stage-4 patients compared with stage-1 patients (*P* = 0.03, Mann-Whitney U test)
